# Aqueous Extracts of the Marine Brown Alga *Lobophora variegata* Inhibit HIV-1 Infection at the Level of Virus Entry into Cells

**DOI:** 10.1371/journal.pone.0103895

**Published:** 2014-08-21

**Authors:** Stephan Kremb, Markus Helfer, Birgit Kraus, Horst Wolff, Christian Wild, Martha Schneider, Christian R. Voolstra, Ruth Brack-Werner

**Affiliations:** 1 Red Sea Research Center, King Abdullah University of Science and Technology (KAUST), Thuwal, Saudi Arabia; 2 Institute of Virology, Helmholtz Zentrum Muenchen, Neuherberg, Germany; 3 Institute of Pharmacy, University of Regensburg, Regensburg, Germany; 4 Carl Zeiss Microscopy GmbH, Goettingen, Germany; 5 Coral Reef Ecology Group (CORE), Leibniz Center for Tropical Marine Ecology (ZMT), Bremen, Germany; 6 University of Bremen, Faculty of Biology and Chemistry, Bremen, Germany; George Mason University, United States of America

## Abstract

In recent years, marine algae have emerged as a rich and promising source of molecules with potent activities against various human pathogens. The widely distributed brown alga *Lobophora variegata* that is often associated with tropical coral reefs exerts strong antibacterial and antiprotozoal effects, but so far has not been associated with specific anti-viral activities. This study investigated potential HIV-1 inhibitory activity of *L. variegata* collected from different geographical regions, using a cell-based full replication HIV-1 reporter assay. Aqueous *L. variegata* extracts showed strong inhibitory effects on several HIV-1 strains, including drug-resistant and primary HIV-1 isolates, and protected even primary cells (PBMC) from HIV-1-infection. Anti-viral potency was related to ecological factors and showed clear differences depending on light exposition or epiphyte growth. Assays addressing early events of the HIV-1 replication cycle indicated that *L. variegata* extracts inhibited entry of HIV-1 into cells at a pre-fusion step possibly by impeding mobility of virus particles. Further characterization of the aqueous extract demonstrated that even high doses had only moderate effects on viability of cultured and primary cells (PBMCs). Imaging-based techniques revealed extract effects on the plasma membrane and actin filaments as well as induction of apoptosis at concentrations exceeding EC_50_ of anti-HIV-1 activity by more than 400 fold. In summary, we show for the first time that *L. variegata* extracts inhibit HIV-1 entry, thereby suggesting this alga as promising source for the development of novel HIV-1 inhibitors.

## Introduction

Despite the implementation of the highly active anti-retroviral therapy (HAART) in 1996, infections with the human immunodeficiency virus 1 (HIV-1) still represent a global threat with more than 34 million infected individuals worldwide and 2.5 million new infections in 2011 (UNAIDS report on the global AIDS epidemic, 2012). Current treatment of HIV-1 infections still has several shortcomings with the emergence of resistant viruses, severe side effects, and high costs being the most pressing issues. Thus, there is an urgent need for novel anti-retroviral therapeutics. In recent years, marine algae have emerged as a rich source of bioactive molecules and have yielded several compounds with remarkable anti-HIV activity, e.g. lectins which inhibit HIV-1 entry and are potential HIV-1 microbicide candidates (reviewed in Huskens and Schols [1]). One of these, Griffithsin (GRFT), was isolated from the marine red alga *Griffithsia sp*., [2] and shows high inhibitory efficacy and safety [3].

Marine brown algal species have also been reported to produce molecules with promising anti-retroviral properties. Among these, sulfated polysaccharides, polyphenols and diterpenes are active constituents [4]–[6]. Examples include Reverse Transcriptase (RT)-inhibiting diterpenes from *Dictyota pfaffii* and sulfated polysaccharides (fucoidans) from *Adenocystis utricularis*, which interfere with early events of HIV-1 replication [7], [8]. Interestingly, as marine macroalgae are consumed by humans on a regular basis in large parts of Eastern Asia, the relatively low rates of HIV infected individuals in these areas may be correlated with the consumption of algae [9]. In fact, a recent proof-of-concept clinical study with two dietary algae indicated improvement of clinical outcomes of HIV-1 infection in terms of CD4 cell count and HIV-1 viral load [10].

*Lobophora variegata* is a common brown alga that is widely distributed in shallow water ecosystems of tropical and subtropical areas, including coral reefs of the Caribbean, the Indian Ocean, and the Red Sea [11], [12]. In coral reefs, *L. variegata* can be an abundant part of the ecosystem and exhibits strong allelochemical defense against potentially deleterious microorganisms [13]. It is able to induce bacterial assemblage shifts as well as sub-lethal effects on reef corals [13], [14]. Kubanek et al. (2003) isolated a cyclic lactone, lobophorilide, showing strong activity against pathogenic and saprophytic marine fungi. Moreover, several studies demonstrated inhibiting effects of chemical constituents of *L. variegata* on several protozoans as well as anti-inflammatory and other health-promoting effects [5], [15]–[20]. *L. variegata* also contains high concentrations of phenolic compounds, mainly bromophenols [4]. Although *L. variegata* is a common and well-studied organism, no specific antiviral effects have been described to date.

The high antibacterial and antiprotist potential of *L. variegata*, led us to investigate whether this organism also displays antiviral activity. Accordingly, we evaluated the capacity of extracts produced from *L. variegata*, specimens collected from coral reefs in different geographical regions (Caribbean and Red Sea) to inhibit HIV-1 infection, using various virus strains. In addition, we used live cell-based and microscopic imaging approaches to characterize mechanisms of activity and potential side effects of algal extracts from *L. variegata*.

## Materials and Methods

### Ethics statement

The study site of Rose Reef (Saudi Arabia) does not fall under any legislative protection or special designation as a marine/environmental protected area. No special permit is required for the inshore coastal, reef, and intertidal areas around Thuwal. The Saudi Coast Guard Authority under the auspices of KAUST University issued sailing permits to the site, which included sample (algae) collection. All necessary permits for the collections at Turneffe Reef (Belize) were obtained for the described study by ALDEBARAN Marine Research&Broadcast (Hamburg, Germany) which complied with all relevant regulations.

### Sample collection and extraction

Thalli of *Lobophora variegata* were collected by SCUBA diving at several locations. Caribbean samples were collected on a sailing trip to Belize with the German small-size research vessel “Aldebaran” in March 2009 at Turneffe reef (17° 16.70′ N, 87° 48.39′ W). Specimens from Northern Red Sea coral reefs were collected on a field trip to Dahab (Sinai, Egypt) in November 2009 at Canyon reef (28° 33.29′ N, 34° 31.247′ E). Samples from Central Red Sea coral reefs were collected at Rose Reef, Saudi Arabia (22° 22.50′ N, 38° 53.83′ E) in April 2012. Directly after collection, any visible contaminations were removed from algal thalli, samples were air dried for 8 to 12 h and stored at −20°C. Prior to extraction, the algal material was ground into a fine powder using liquid nitrogen. Aqueous and methanolic extracts were prepared by addition of 1 ml distilled water or methanol to 100 mg of powdered algal material. Samples were briefly vortexed and extracted at 4°C overnight. Subsequently, samples were centrifuged at 13,000 g for 30 min to remove particulate material and then stored at −20°C until further use. Unless otherwise stated, the aqueous extract prepared from full-sunlight exposed thalli of *L. variegata* was used for all experiments.

In order to analyze the solubility of potentially active compounds of the aqueous *L. variegata* extract in organic solvents, liquid/liquid extraction was performed. Briefly, 3 ml of extract were mixed with 3 ml of hexane or chloroform, shaken for 2 min and centrifuged at 13,000 g for 20 min for efficient phase separation. The resulting solvent phases were dried in the rotation evaporator and re-suspended in ultrapure water (Chromasolv, Sigma Aldrich, Taufkirchen, Germany).

### Cell culture

HeLa cells, HEK 293T cells, and the HIV-1 indicator cell lines (LC5-RIC and LC5-RIC-R5) were kept under standard conditions at 37°C in 5% CO_2_ in Dulbecco’s modified Eagle medium (DMEM containing GlutaMAX-1; Gibco, Darmstadt, Germany) or very-low-endotoxin(VLE)-RPMI 1640 medium (Biochrom AG, Berlin, Germany) supplemented with 10% fetal bovine serum (Biochrom AG) and 1% antibiotic-antimycotic solution (Gibco). In order to maintain stable reporter and CD4 receptor expression of LC5-RIC/LC5-RIC-R5 reporter cells, 0.74 mg ml^−1^ Geneticin (G418 sulfate; PAA Laboratories, Pasching, Austria) and 0.13 mg ml^−1^ hygromycin B (PAA Laboratories) were added to the cell culture medium at every second passage.

### Virus stock preparation

Virus stocks were produced in HEK 293T cells by transfection with the following infectious molecular clones: pLAI.2, pNL(AD8) (HIV-1 AD8 Macrophage-Tropic R5), HIV-1 p7324-1 (multidrug resistant molecular clone with patient-derived mutations in Reverse Transcriptase encoding sequences) and pBR-NL4-3 V92th014.12-IRES-eGFP (HIV-1 NL4-3 Gag-iGFP-reporter virus,[21]) Briefly, HEK 293T cells were seeded in 6-well plates and transfected with 1 µg of the corresponding plasmid DNA per well using 3.8 µl of FuGene HD transfection reagent (Roche), following the manufacturer’s instructions. After 72 h, the supernatant was harvested, centrifuged for 5 min at 2,000 g, aliquoted, and frozen at −80°C. Pseudo-typed viruses harboring the glycoprotein of the Vesicular Stomatitis Virus (VSVg) were prepared by co-transfection of HEK 293T cells with 0.5 µg of the HIV-1 env-defective molecular clone pNL4–3Δenv and 0.5 µg of pMD2.G (Addgene, MA) for expression of VSV-G. The HIV-1 strain HIV-1 KIII was prepared from supernatants of the HIV-1_IIIB_ producer cell line KE37.1-IIIB. Briefly, KE37.1-IIIB cells were adjusted to 8×10^5^ per ml and cultured for 48 h. Virus-containing culture supernatants were harvested by centrifugation at 2,000 g, passed through a 0.45 µm pore-sized filter (Sartorius, Goettingen, Germany), aliquoted and frozen at −80°C. The primary HIV-1 isolate HIV-1 PAT891 was isolated from cerebrospinal fluid (CSF) of an HIV-1 infected individual as described elsewhere [22]. Virus preparations were quantified by determining p24 levels (Gag-p24 enzyme-linked immunosorbent assay; Applied Biosystems, Carlsbad, CA), according to the manufacturer’s instructions. In addition, viral preparations were tested on LC5-RIC cells in order to determine levels of infectious viruses. The volumes of virus stocks that were used for infection of LC5-RIC cells were determined according to the following critera: (i) more than 100-fold increase of the relative fluorescent signal by HIV-1 infection, (ii) relative signal induction levels below the plateau, and (iii) a reduction of the relative MTT signal by less than 10%. [22].

### Standard assay setup for testing of HIV-1 inhibitory activities

A description of the full assay procedure can be found elsewhere [22]. Briefly, LC5-RIC cells were seeded into 96-well plates (µCLEAR-Plate Black; Greiner Bio-One, Kremsmuenster, Germany) at a density of 10^4^ per well at 24 h prior to infection. *L. variegata* aqueous extracts were tested in serial dilutions in triplicates. Stock solutions of aequous *L. variegata* extracts, reference compounds and virus preparations were diluted with cell culture medium. For treatment and infection, 100 µl of compound solution and 20 µl of diluted virus inoculum were added to each test well, with each extract concentration tested in triplicate wells. Plates were incubated for 48 h after virus addition under standard conditions at 37°C in 5% CO and subsequently tested for fluorescent reporter signal intensity with a fluorescence microplate reader (Tecan infinite M200 (Tecan, Crailsheim, Germany)) at wavelengths 552 nm for excitation and 596 for emission. The influence of the extracts on the viability of LC5-RIC cells was determined by a standard MTT test [23] directly after reporter signal measurement. Cell cultures were incubated with 50 µg of MTT solution (Sigma-Aldrich, Taufkirchen, Germany) in 100 µl of culture medium for 2 h under standard culture conditions. After removal of MTT solution, cells were lysed by addition of 100 µl of lysis solution (10.0% [wt/vol] SDS and 0.6% [vol/vol] acetic acid in dimethylsulfoxide [DMSO]). MTT formazan concentrations were determined by an ELISA plate reader (Tecan Infinite M200) at a test wavelength of 570 nm and a reference wavelength of 630 nm. Values for treated HIV-1-infected cultures were related to those of untreated, HIV-1-infected, cultures in the same plate.

### RNA and DNA quantification

For the quantification of viral RNA and DNA in infected LC5-CD4 cells, cells were seeded in 12-well plates. 24 hours later medium was replaced and cells were treated with either 65 µg/ml *L. variegata* aqueous extract or PBS and exposed to HIV-1_LAI_. For the RNA-quantification, 200 nM Efavirenz was added to each sample to inhibit RNA-turnover by HIV-1 reverse transcriptase. Samples for RNA and DNA quantification were collected 4 or 24 hours p.i., respectively. After washing of cells with PBS before (three times) and after (twice) trypsination, RNA and DNA were isolated using the RNeasy kit or the DNA Mini (QIAGEN) according to the manufacturer’s manuals. RNA and DNA quantification was performed as described in Helfer *et al*. (2014) [24]. Briefly, RNA levels were quantified by relative qPCR with specific primers for HIV-1 and calculation was performed with the −2^−ΔΔCT^ method. Expression of RNA-Polymerase II was used as reference. For determination of HIV-1 DNA loads, an absolute quantification was done by qPCR using the TH4-7-5 cell line as external standard. β-globin was used as reference gene (Kabamba-Mukadi *et al.,* 2005) [25].

### PBMC Assay

Peripheral blood mononuclear cells (PBMCs) were prepared from whole blood of healthy donors (obtained from the blood bank of the German Red Cross, Munich, Germany) according to a standard protocol (Current protocols in immunology; 7.1.1–7.1.8 April 2009, Supplement 85). 72 h prior to infection, PBMC (pools of cells from 4 donors) were stimulated by the addition of Interleukin-2 (50 units per ml of cell culture medium; Sigma-Aldrich, Taufkirchen, Germany). For infection experiments, 5×10^5^ cells per well were seeded into 96-well plates. Treatment and infection of PBMC was carried out according to the standard assay setup as described above by using the HIV-1 LAI strain for infection. Four days after infection, 20 µl of supernatant from each well was transferred to LC5-RIC indicator cells in order to assay the production of infectious HIV-1 progeny from treated or untreated PBMC. The reporter signal intensity of the LC5-RIC indicator cells was measured at 48 h after addition of PBMC supernatants. Vitality of PBMCs was determined by an MTT test as described for LC5-RIC cells.

### High Content Analysis

HeLa cells were transferred into 96-well plates, treated with different concentrations of *L. variegata*-extract for 24 h, then fixed and nuclei stained with Hoechst 33342 and β-tubulin stained with β-tubulin Alexa Fluor 488 (Life Technologies, Darmstadt, Germany). Images were acquired with a ZEISS Cell Observer (Carl Zeiss, Jena, Germany) system and a 10x Plan-NEOfluar objective with filters sets No. 49 (360/40; FT 400; BP 460/50 and 38 (475/40; FT 500; BP 530/50). Images were analyzed using the *Physiology* Analyst of the ASSAYbuilder module of AxioVision 4.6. Following the manufactureŕs protocol, nuclei were used to automatically detect cells. A ring mask around the nucleus was generated and used to detect β-tubulin fluorescence. The cell numbers and the nucleus shape of individual cells was determined for at least 4 randomly selected fields of view per well. The number of untreated control cells was set to 100%, and a treatment-caused alteration was set in relation to this. The average nuclear form factor of the control cells was used as a reference for a normal state. Increased form factor represents a tendency towards fragmented and irregular nuclei, which is usually an indicator of apoptosis, whereas a decreased form factor typically results from rounded up cells, indicating necrosis.

### Single Cell Analysis

By controlling locations of adhesive (fibronectin) and non-adhesive areas (PEG) on a coverslip, micro-patterns are generated and result in a highly reproducible and polarized cell organization. Using this technique, many thousands of living cells can be positioned on a single coverslip chip, and cellular feature analysis can subsequently be performed with optimal reproducibility and accuracy. HeLa cells were plated on CYTOO chip (Cytoo, Grenoble, France) coverslip with Y-shaped fibronectin micro-patterns. Cells were either left untreated (control) or treated for 24 h with *L. variegata* aqueous extract at a final concentration of 2000 µg ml^−1^ in culture medium. Subsequently, cells were fixed and stained for DNA (Hoechst33342), actin (Alexa Fluor 488 Phalloidin, Life Technologies) and membrane (Wheat Germ AgglutininTexasRed, Life Technologies). Cells were imaged with a ZEISS Cell Observer System and a 20x Plan-APOchromat objective with filters sets No. 49 (blue), 38 (green) and 43 (red).

### TOA

Time-of-addition (TOA) assays were carried out using a slightly modified standard assay setup in 96-well plates using 100 µl of compound solution and 20 µl of diluted virus inoculum. Virus (HIV-1 LAI) was added to LC5-RIC cultures at time point 0. Reference inhibitory compounds (Griffithsin (GRFT), T-20, AZT (Zidovudine) and Efavirenz (EFV), all obtained from the NIH AIDS Reagent Program) and the aqueous *L. variegata* extract were added to the cultures at different time points after addition of virus preparations to final concentrations of 2x EC_50_ in each well. At least 10 different time points were evaluated in triplicates for each compound. Cultures were incubated for 48 h and subsequently analyzed for reporter signal intensity as described above.

### Analysis of HIV-1 attachment

HIV-1 attachment assays were performed with LC5-RIC-R5 cells and HIV-1 NL4-3 Gag-iGFP reporter virus in the presence of the fusion inhibitor T20 (100 nM) to promote attachment of virus particles to cells. Cells were plated on 24×24 mm cover slips in 6-well plates. After 24 h, the culture medium was replaced with fresh medium containing T20 (50 nM) and investigational inhibitors (i.e. 250 µg ml^−1^ *L. variegata* extract, or 50 nM Griffithsin) and virus inoculum (∼8 pg p24 per cell). Control samples lacked investigational inhibitors in the medium and included samples with virus inoculum ( = virus control) or without virus (background control). The cells were then incubated at 37°C for 4 hours, washed once with PBS and treated with 2% PFA containing DAPI for 20 min at room temperature. The cells were then washed again with PBS, and cover slips were fixed onto glass slides with Mowiol (Polysciences, Inc.) overnight at room temperature. Analysis was carried out by fluorescence microscopy (Nikon TiE equipped with Perkin Elmer UltraView Vox System). Exposure-times: GFP: 100 ms, DAPI: various.

### Calculation of values and curve fits

Curve fits and EC_50_ calculations were performed by SigmaPlot, version 11.0 (Systat Software, Chicago, IL) or Prism, version 4 (GraphPad Software, La Jolla, CA). Statistical analysis of data was carried out with the GraphPad Prism program. Significances of differences between data sets were determined by calculating two-tailed P values using the Mann-Whitney-U test.

## Results

### Aqueous extracts of *Lobophora variegata* specimens from different geographical origins inhibit HIV-1 replication

**Figure 1:**
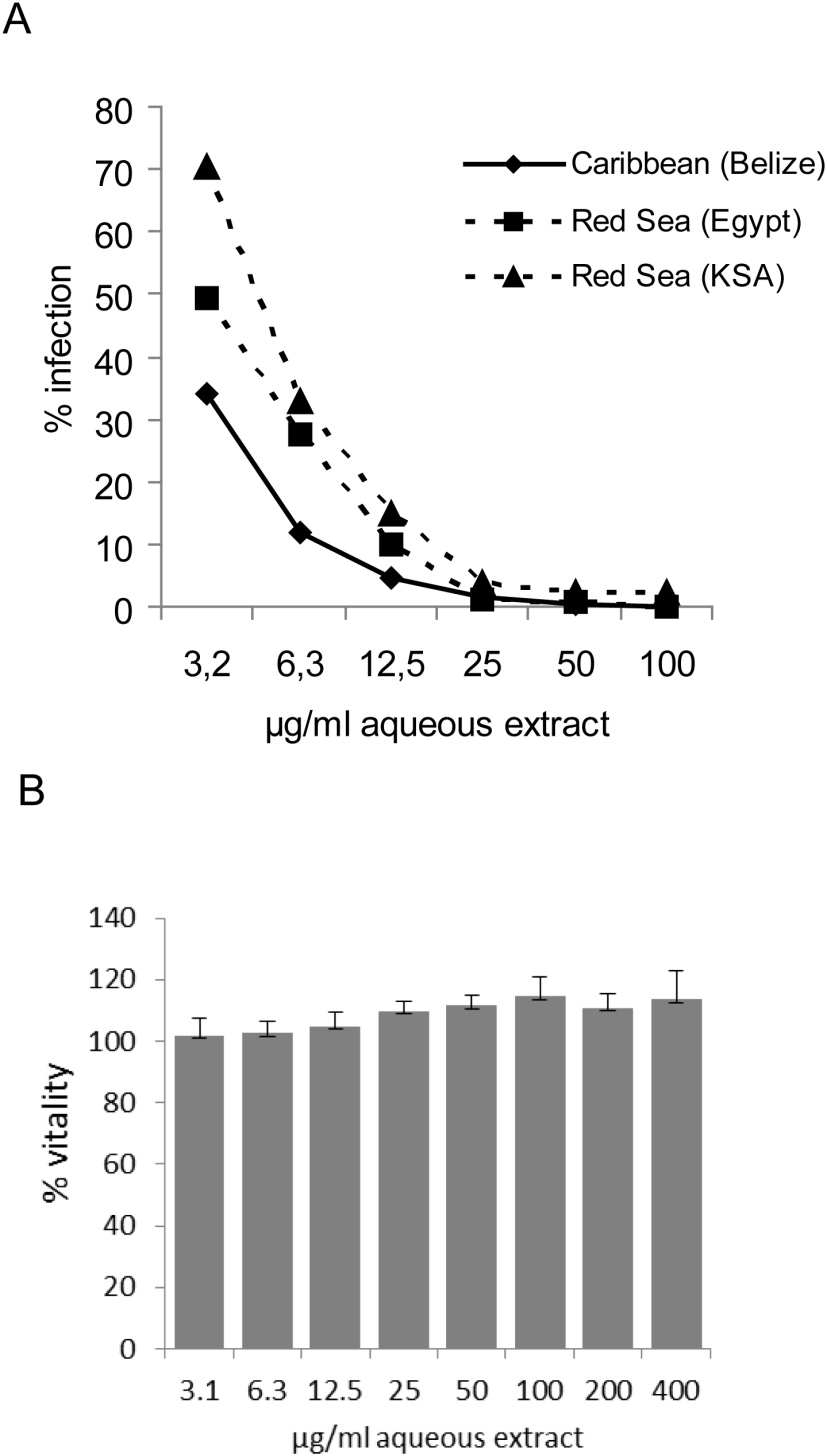
Dose-dependent inhibition of HIV-1 replication and vitality of reporter cells. **A**: Dose-dependent inhibition of HIV-1 (strain HIV-LAI) replication in LC5-RIC reporter cells by aqueous extracts from specimens collected at various geographical locations including the Caribbean (Turneffe Reef, Belize), northern Red Sea (Sinai, Egypt, Canyon Reef) and the central Red Sea (Rose Reef, Kingdom of Saudi Arabia). Cells were treated with serial dilutions of the aqueous *L. variegata* extract (ranging from 3.2 up to 100 µg/ml) in triplicates and exposed to the HIV-1 LAI strain. **B**: Effects of different concentrations of *L. variegata* extract (collected from Turneffe Reef, Belize) on viability of indicator cells were measured by the colorimetric MTT assay. LC5-RIC reporter cells were treated with serial dilutions (ranging from 3.1 up to 400 µg/ml) of the aqueous *L. variegata* extract for 48 hours. Data are given relative to untreated cells (100%).

Several aqueous extracts of *Lobophora variegata* collected from various geographical origins were tested in a cell-based full-replication assay for anti-HIV-1 activity. All aqueous extracts inhibited HIV-1 replication in a clearly dose-dependent manner. (Figure 1A). Liquid/liquid extractions of the *L. variegata* extracts with organic solvents revealed anti-HIV-1 activity solely in the aqueous and not in the organic phases, pointing to a highly hydrophilic nature of the active components (data not shown).

### *L. variegata* aqueous extracts show only minor toxic effects at even high concentrations

**Figure 2:**
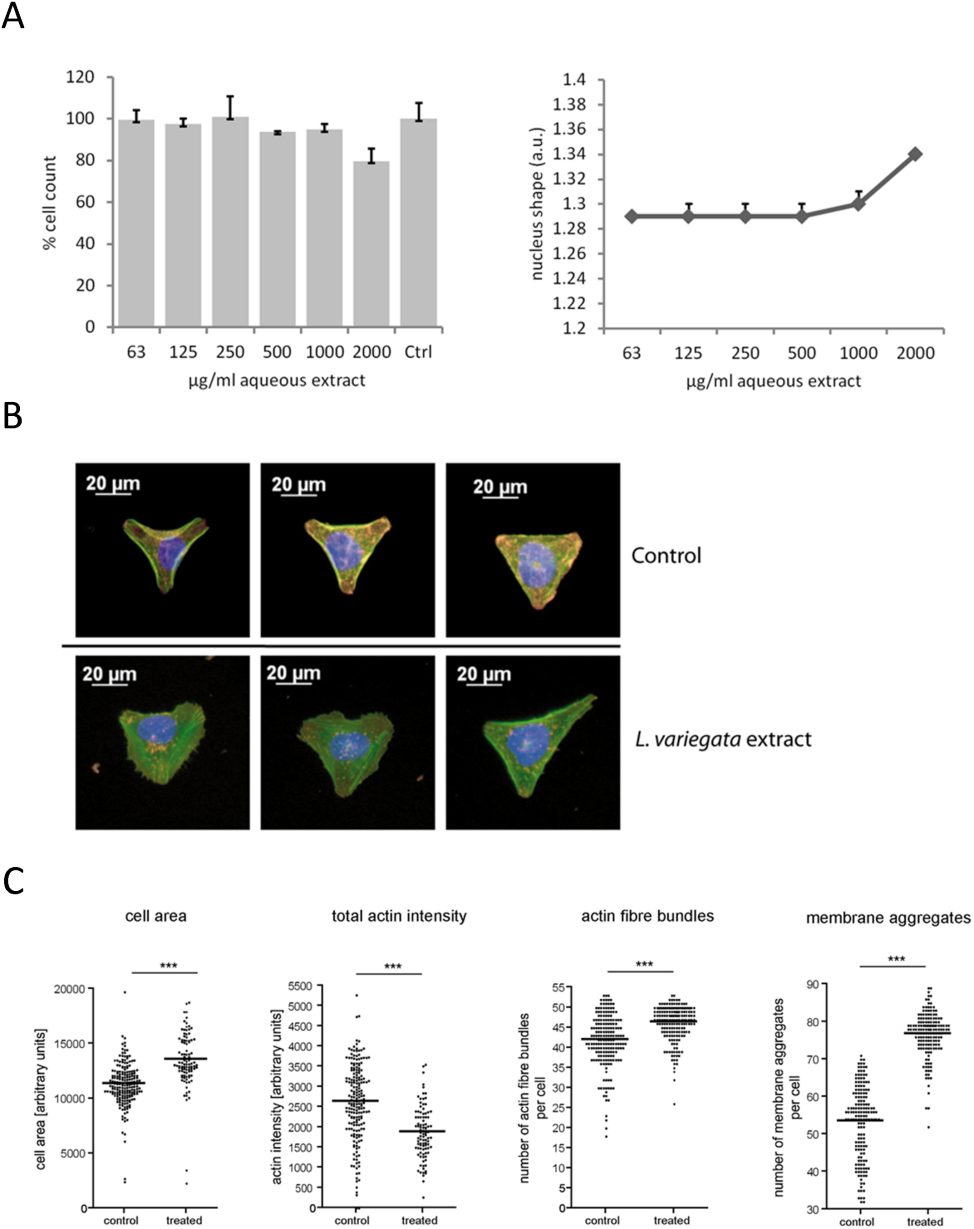
Effects of treatment with different concentrations of *L. variegata* extracts on multiple cellular parameters. **A**: Effects of high extract concentrations on cell loss (left) and nuclear shape of HeLa cells as an indicator of either apoptosis or necrosis (right). **B**: CYTOO chip single cell assay for morphometry: microscopic images of HeLa cells spotted on fibronectin Y-patterns either control- (top row) or extract- (bottom row) treated (50 mg ml^−1^, nuclei in blue, actin in green, membrane in orange/red). **C**: Results of automatic feature extraction by High Content Analysis done with approx. 150 cells per experiment and treatment. Asterisks indicate statistical significance (***p<0,001).

Effects of *L. variegata* extracts on cells were first assessed by MTT assay**,** which measures cellular metabolic activity and is often used to assay cell viability. Viability of the indicator cells was not affected by treatment with *L. variegata* extracts at concentrations up to 400 µg ml^−1^, which exceeded the concentration required for full HIV-1 inhibition (approx. 50 µg ml^−1^) (Figure 1B). Even a long-term exposure of Hela cells with a single dose of 400 µg ml^−1^ for up to seven days did not result in any detectable toxicity (Fig. S1 in File S1). Toxic effects were also studied in HeLa cells treated with extract concentrations up to 2 mg ml^−1^ by High Content Analysis of cells with stained nuclei and microtubuli. Whereas no significant effects on cell cycle state (ploidity) and β-tubulin were observed (data not shown), mild cell loss of approximately 20% and a slight induction of apoptosis was observed in cells treated with the highest extract concentration (Figure 2A).

Next, effects of the *L. variegata* aqueous extract were analyzed on the single cell level. Microscopic images clearly depicted pronounced changes of several cellular features at higher extract concentrations, including a reduced membrane signal, re-arrangement of actin filaments, and a less well-defined Y-shaped morphology (Figure 2B). Automated cell feature extraction by High Content Analysis revealed a significant increase of cell size (mean cell area), reduction of mean actin intensity, increase in the number of actin fiber bundles, and substantially increased number of membrane aggregates (Figure 2C).

### *L. variegata* aqueous extract is active against a panel of HIV strains and in primary cells

In order to further characterize the anti-HIV-1 potential of aqueous *L. variegata* extracts, we tested a panel of HIV-1 variants consisting of laboratory strains with different cellular tropisms, a multi-drug resistant HIV-1 strain with patient-derived reverse transcriptase mutations, a primary HIV-1 strain derived from the cerebrospinal fluid of an HIV-1 infected patient (Kremb et al., 2010) and HIV-1 particles pseudotyped with the G-Protein of the *Vesicular stomatitis virus* (Table 1). *L. variegata* extracts inhibited infection by the two X4-tropic (lymphotropic) strains, HIV-1_LAI_ and HIV-1_KIII_ as well as the R5-tropic (macrophage-tropic) strain, HIV-1_AD8_. The corresponding EC_50_ values ranged from 2.6 to 8.1 µg extract per ml and did not correlate with virus tropism. Moreover, we found a potent inhibitory activity against the multi-drug-resistant HIV-1 strain and the primary HIV-1 virus isolate. Furthermore, *L. variegata* extracts also inhibited infection by the VSV-G pseudotyped HIV-1 particles with an EC_50_ value similar to those obtained for native HIV-1 strains.

**Table 1:** Evaluation of anti-HIV-1 activity of aqueous *L. variegata* extract activity with a panel of HIV-1 strains and LC5-RIC indicator cells.

Virus strain	characteristics	EC50	r2
HIV-1 LAI	X4 tropic	8.12	1.0000
HIV-1 KIII	X4 tropic	3.64	1.0000
HIV-1 AD8	R5 tropic	2.62	0.9980
HIV-1 p7324–1	multi-drug-resistant	7.77	0.9989
HIV-1 PAT891	primary isolate	5.38	0.9982
HIV-1 VSVg pp	VSV envelope	4.93	1.0000

The panel includes viruses using the chemokine co-receptor CXCR4 (X4 tropic) or CCR5 (R5 tropic), a multi-drug-resistant HIV-1 variant with patient-derived *reverse transcriptase* mutations, a primary HIV-1 isolate isolated from cerebrospinal fluid, as well as HV-1 virus particles bearing the G-protein of the vesicular stomatitis virus in the envelope. EC_50_ values (µg aqueous extract per ml cell culture medium) were calculated by SigmaPlot and R-squared parameters are given.

**Figure 3:**
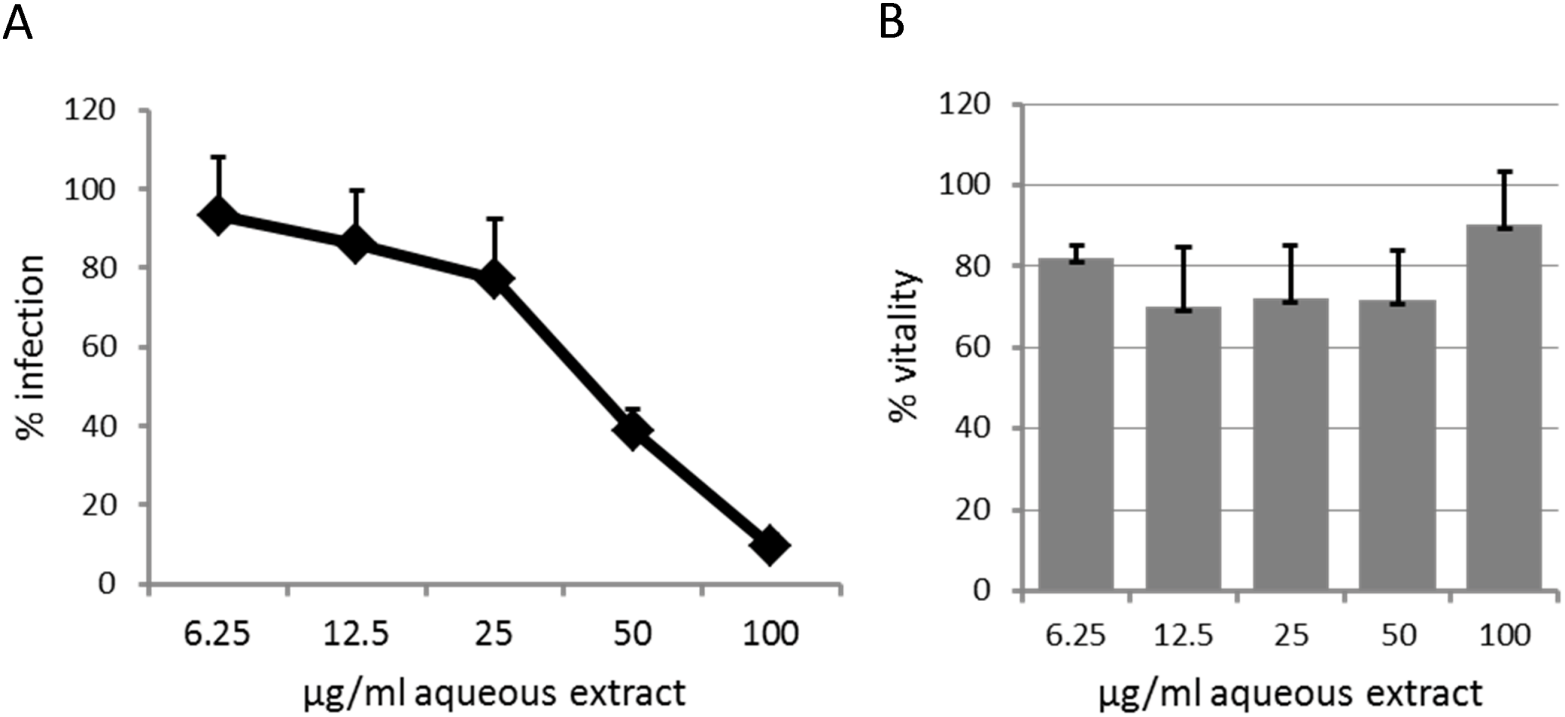
Effects of the *L. variegata* extract on HIV-1 infection of primary cells. **A**: Dose-dependent inhibition of HIV-1 replication in primary cells treated with *L. variegata* aqueous extract. Peripheral blood mononuclear cells (PBMC) were isolated from whole blood and exposed to the lymphotropic variant HIV1-LAI. Pooled PBMCs were stimulated by treatment with IL-2 for 72 hours, followed by the addition of virus inoculum (HIV-1 LAI) and diluted extracts and continued incubation for 4 days under standard cell culture conditions. The production of infectious HIV-1 progeny was titrated on LC5-RIC indicator cells. **B**: Vitality of HIV-1-infected PBMC at corresponding extract concentrations.

As HeLa cells are not the natural target cells of HIV-1, we further tested the potency of *L. variegata* aqueous extract to protect primary cells from HIV-1 infection. We found that *L. variegata* aqueous extract also inhibited HIV-1 replication in peripheral blood mononuclear cells (Figure 3A) with a moderate decrease of cell vitality at intermediate extract concentrations (Figure 3B).

### *L. variegata* aqueous extract inhibits HIV-1 infection at an early stage of virus replication

**Figure 4:**
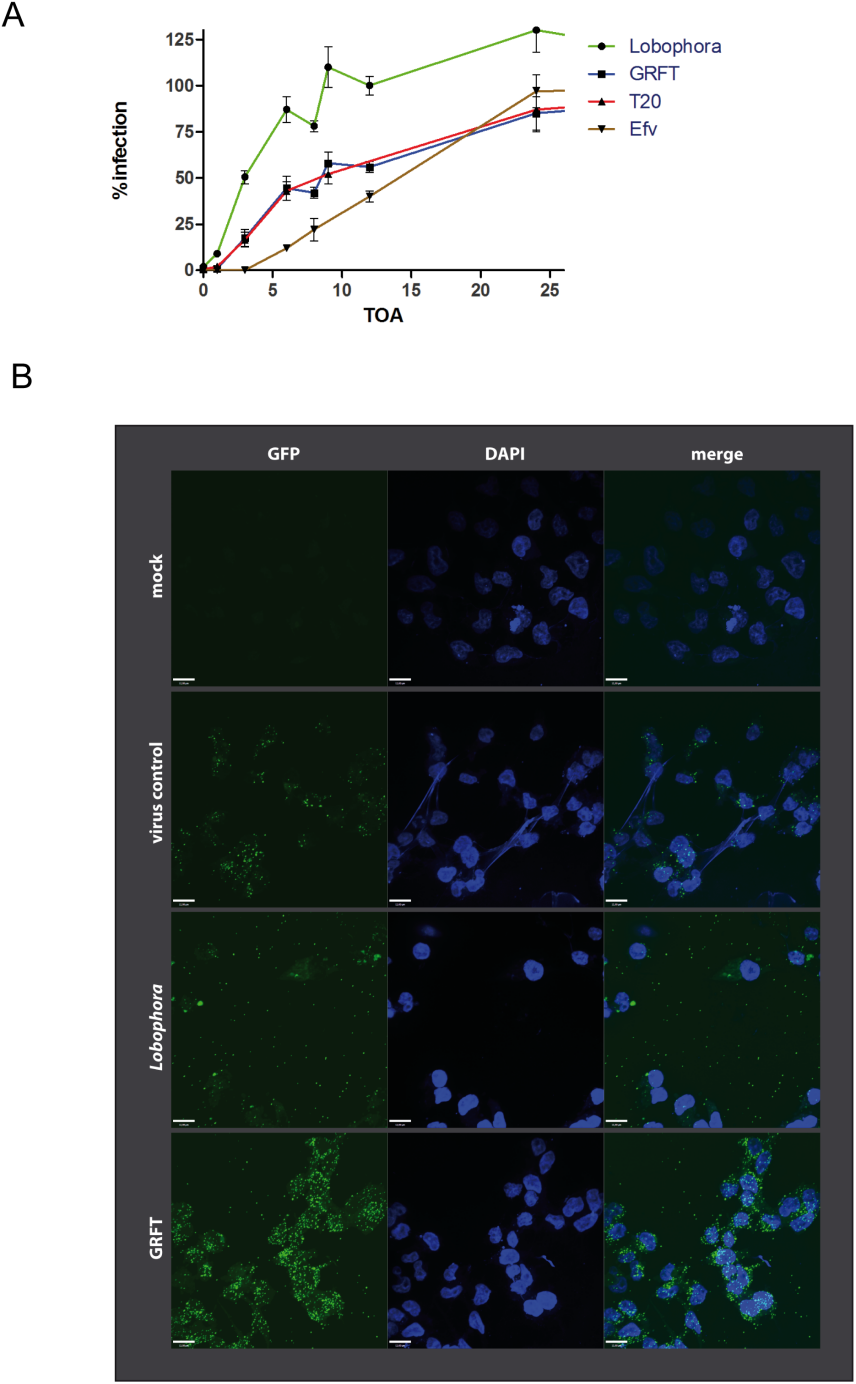
Analysis of the mechanism-of-action of the *L. variegata* extract. **A**: Time-of-compound addition dependent inhibition of HIV-1 replication. HIV-1 indicator cells were treated with various reference compounds targeting approved steps of HIV-1 replication (GRFT: virus attachment; T-20: virus/cell fusion; AZT: reverse transcription, competitive; EFV: reverse transcription, non-competitive) and the *L. variegata* aqueous extract at concentrations of 2× EC50. All LC5-RIC indicator cell cultures were exposed to HIV-1 LAI. at time point 0 with test and reference compounds were added to the cells at different time points. At least 10 different time points in triplicates were measured for each compound. **B**: Analysis of HIV-1 attachment using GFP-labeled HIV-1 particles. HIV-1 indicator cells were exposed to the HIV-1 NL4-3 Gag-iGFP reporter virus for 4 hours in the presence of the fusion inhibitor T20 and test compounds (250 µg/ml aqueous *L. variegata* extract or 50 nM Griffithsin). Nuclei were stained with DAPI.

**Figure 5:**
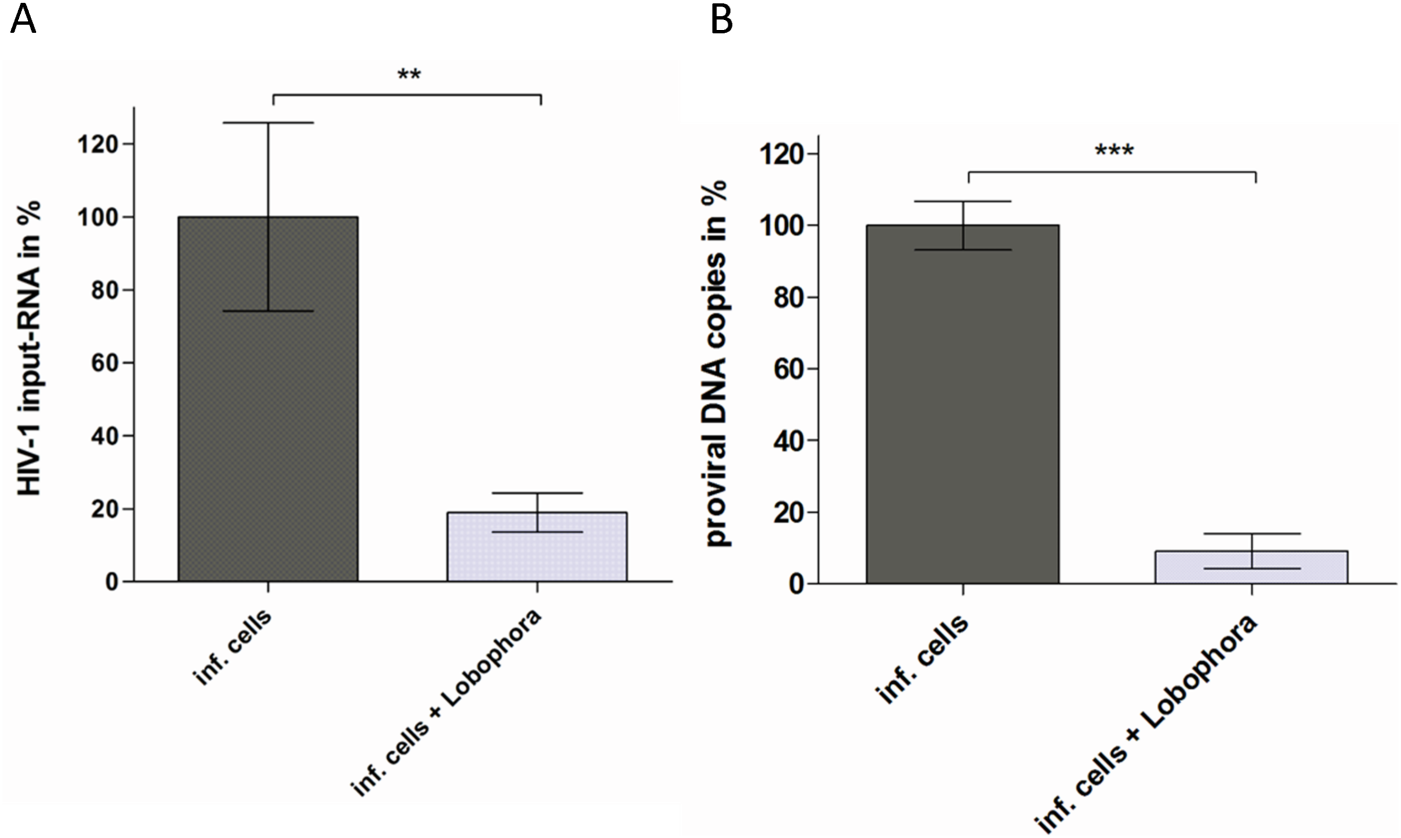
*L. variegata* aqueous extract inhibits HIV entry. **A.** Reduction of input HIV-1 viral RNA levels by treatment with *L. variegata* aqueous extract. LC5-CD4 cells were infected with HIV-1 in the absence or presence *of L. variegata* aqueous extract. 4 hours p.i. cells were washed extensively to remove inoculum and subsequently total RNA was isolated. After reverse transcription, qPCR was performed to quantify viral RNA levels. HIV-1 transcript levels were normalized to RPII levels. Means and standard deviations are shown in relation to untreated/uninfected cells in percent. **B.** Reduction of HIV-1 DNA loads by treatment with *L. variegata* aqueous extract. LC5-CD4 cells were infected with HIV-1 in the absence or presence of *L. variegata* aqueous extract. 24 hours later, cells were washed extensively to remove inoculum RNA isolated and cDNA produced from RNA. To quantify viral DNA loads, absolute qPCR was performed. Means and standard deviations are shown in relation to untreated/uninfected samples in percent.

Our next aim was to characterize the mode-of-action by which the *L. variegata* extract inhibits HIV-1 replication. We first employed a time-of-addition assay [22] to identify which steps of the HIV-1 replication cycle are affected by the *L. variegata* extract (Figure 4A). This assay revealed that *L. variegata* extract clearly inhibits an early step in the HIV-1 replication cycle which is different from and precedes the steps targeted by the entry inhibitor Griffithsin, the fusion inhibitor T-20 and the reverse transcription inhibitor Efavirenz. To evaluate the effect of *L. variegata* extract on HIV-1 attachment behavior, GFP-labelled virus particles were incubated with HIV-1 indicator cells in medium containing investigational inhibitors (i.e. *L. variegata* extract or Griffithsin) and the distribution of HIV-1 particles was analyzed by spinning-disc confocal microscopy (Figure 4B). All samples also contained the fusion inhibitor T20 to prevent virus uptake. Virus particles associated predominantly with host cells in virus-containing samples lacking investigational inibitors (i.e. virus control in Figure 4B). Attachment of virus particles was increased even further by treatment with Griffithsin, which is in agreement with the reported enhancement of virus binding to the CD4-receptor by Griffithsin [26]. In contrast, treatment with *L. variegata* extract led to more random distribution of virus particles with occurrence of virus particles in cell-free areas of the slide, indicating arbitrary attachment of the virus particles to non-cellular surfaces. These results suggest that *L. variegata* extracts may change the attachment behavior of virus particles, possibly promoting their attachment to unspecific surfaces. Additional experiments were conducted to validate the inhibition of HIV-1 at an early stage of the viral replication cycle. Quantification of intracellular HIV-1 RNA levels in HIV-1 target cells (LC5-CD4) at 4 hours post infection showed a substantial reduction of viral RNA levels in cells treated with the *L. variegata* extract (Figure 5A). Similarly, quantification of proviral copies revealed a clearly reduced viral load in extract-treated cells (Figure 5B).

### Environmental conditions affect anti-HIV-1 activity of the *L. variegata* aqueous extract

**Figure 6:**
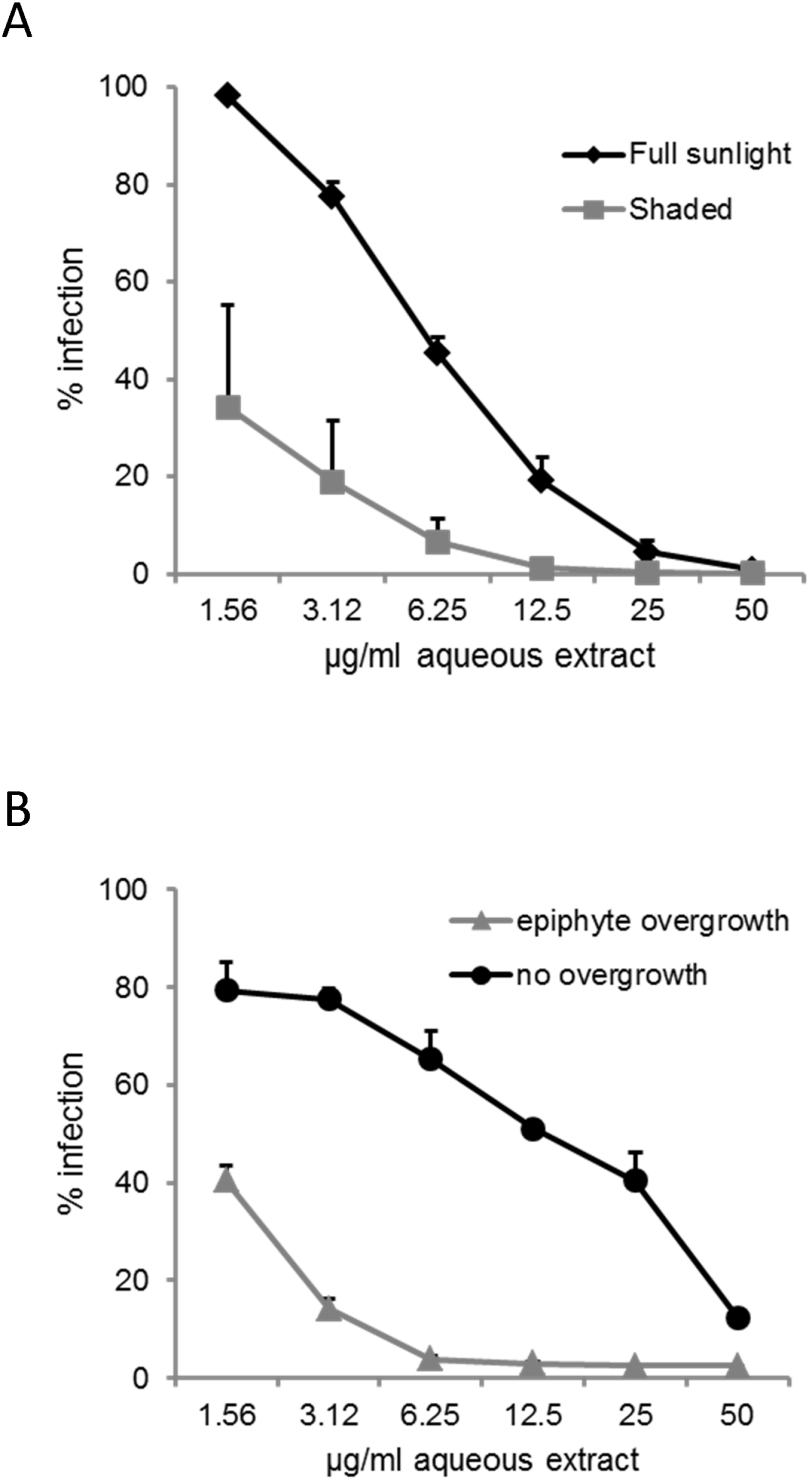
Effects of different growth conditions of *L. variegata* on the HIV-1 inhibitory potential. **A**: Comparison of extract activities of specimens from the Northern Red Sea, collected from sunny or shady locations. **B**: Comparison of extract activities of specimens from the Caribbean with or without overgrowth by epiphytes. Epiphytes were removed before extraction of *L. variegata*. All extracts were tested on LC5-RIC reporter cells using the HIV-1 strain HIV-LAI.

To test whether growth conditions of *Lobophora* affect its HIV-1 inhibitory potency we compared the activities of *Lobophora* samples collected from various settings. All samples showed anti-HIV-1 activity, regardless of their geographical origin (Caribbean and Red Sea). However, specimens from the same geographical origins that were exposed to different environmental conditions showed different potencies of anti-HIV-1 activity. Thus, extracts of Northern Red Sea specimens collected from a shady location showed higher anti-HIV-1 activity than extracts of specimens from a sunny location. (Figure 6A). Furthermore, extracts of Caribbean specimens that had been overgrown by epiphytes showed higher anti-HIV-1 activity than extracts of samples free of epiphytes. (Figure 6B).

## Discussion

In this study, a combination of a cell-based HIV-1 full replication assay and imaging-based techniques was used to identify and characterize anti-HIV-1 activities in a crude aqueous extract of the brown alga *Lobophora variegata*.

All aqueous extracts of *L. variegata* showed potent and dose-dependent inhibition of HIV-1 replication, although the specimens used in their production originated from different and far apart geographical locations, (i.e. Atlantic (Caribbean) and Indo-Pacific (Red Sea). This suggests that all *L. variegata* specimens are capable of inhibiting HIV-1 activity by a common principle.

Anti-HIV-1 activity of *L. variegata* was detected exclusively in aqueous but not in organic phases of the extract, indicating a highly polar nature of the active principle. Similarly, other algae-derived molecules with high anti-retroviral activity, such as sulfated polysaccharides and the lectin Griffithsin, were also identified in aqueous extracts [2], [6]. In contrast, anti-protozoal activities of *L. variegata* occurred exclusively in organic extracts. This indicates that anti-protozoal and anti-HIV-1 activities are mediated by different, unrelated components of *L. variegata*.

Evaluation of cellular toxicity of extracts by MTT assay, which is an indicator of cell metabolism [27], revealed no toxic effects of the extract on the HIV-1 indicator cells even at the highest concentrations. To evaluate effects of *L. variegata* on other cellular parameters we used High Content Analysis which allows simultaneous analysis of multiple cellular parameters. This technology, revealed no impairment of the cell cycle at any concentration (data not shown), at any concentration. However, changes in nuclear shapes associated with apoptosis, increased cell areas and changes in the actin cytoskeleton were apparent at the highest extract concentration of 2 mg ml^−1^. Furthermore, treatment with *L. variegata* extract at this high concentration had a relatively pronounced effect on the plasma membrane, suggesting that the *L. variegata* aqueous extract also contains a membrane-active constituent. These cellular effects only occur at much higher extract concentrations than those required for anti-HIV-1 activity and can most probably be attributed to other compounds. However, to further improve the safety of *L. variegata* extracts for humans both activities should be separated.

*L. variegata* extracts inhibited replication of all tested virus strains including HIV-1 strains with different cellular tropisms, a multi-drug resistant HIV-1 strain and a primary HIV-1 isolate obtained from an infected individual. In addition, anti-HIV-1 activity was confirmed with primary human HIV-1 target cells (PBMC). Together these data support the ability of the *L. variegata* aqueous extract to inhibit HIV-1 infection under physiologically relevant conditions.

To learn more about the mechanism-of-action by which the extract targets HIV-1 replication, we employed a strategy of time-dependent addition of the test compound to infected cells. This method was recently demonstrated to be adaptable to crude biological extracts [22]. The resulting inhibition curve clearly points to an interference of the *L. variegata* aqueous extract with a very early step of virus replication. A substantial reduction of viral RNA levels and proviral copies in infected cells treated with the *L. variegata* extract further support this hypothesis. We next analyzed the effect of the extract on the attachment of GFP-labeled HIV-1 particles to target cells by microscopic imaging. The observed unspecific immobilization of viral particles on surfaces including non-cellular parts indicates a direct interaction of extract compounds with HIV-1 virions. Although viral particles can be found attached to cells, viral entry is obviously impaired. This may either be related to a virucidal effect or to an immobilization of the virus on the cell membrane making it impossible for the virus to further proceed in its replication cycle. The pronounced effect of the extract on the plasma membrane at high extract concentrations, including a reduced membrane signal and an increase of membrane aggregates, might be related to the presence of membrane-active compounds. These could interfere with the viral membrane leading to the observed immobilization and impaired viral entry into target cells. In addition, the strong inhibition of virions pseudotyped with a non-HIV-1 glycoprotein (VSV-g) makes a specific interaction of extract compounds with the HIV-1 glycoproteins unlikely and argues in favor of the viral membrane as the target structure. This inhibitory mechanism is different from the well-characterized HIV-1 entry inhibitors T-20 and Griffithsin or the plant-derived triterpene glycyrrhizin which may reduce the fluidity of the plasma membrane leading to an inhibition of virus/cell fusion [28], [29]. Thus, the anti-viral effect of the *L. variegata* aqueous extract may represent a new inhibitory principle and should be characterized in more detail.

*L. variegata* inhabits a wide range of ecological settings and can have strong allelochemical effects on its environment [13], [14]. It is likely that different growth conditions may have effects on the chemical composition of the thalli and thus on anti-viral activity. Our results clearly showed pronounced differences in anti-HIV-1 activities of algae from different environmental settings. Apparently, environmental factors such as light conditions or overgrowth by epiphytes influence anti-retroviral activities of specimens. Environmental stress generally leads to the induction of diverse protective molecules in algae [30]. These include polyphenols, sulfated polysaccharides, or proteins and are used as sunscreens, scavengers of reactive oxygen species (ROS), deterrents, or anti-microbial agents by these organisms [30], [31]. Thus, it is possible that the anti-HIV-1 activity observed in the aqueous extract may be part of the stress response of this alga.

In summary, our results reveal for the first time a strong and broad anti-retroviral activity of the common brown macroalga *L. variegata* and suggest a direct interaction of extract constituents with the viral particles. These findings further complete the rich anti-microbial spectrum of this algal species and suggest its extracts as a promising source for the development of novel HIV-1 inhibitors.

## Supporting Information

File S1 **Figure S1 and supplemental experimental procedures. Figure S1: Time-dependent effect of the** ***L. variegata*** **aqueous extract on the viability of Hela cells.** A time series of the effect of the *L. variegata* aqueous extract on viability of Hela cells was determined by a colorimetric XTT assay. Hela cells were treated with a fixed concentration of 400 µg/ml of the extract for time periods of one day up to 7 days. Data are given relative to untreated cells measured at the same time point (100%).
(DOCX)
