# Supplementary material for: Aqueous Extracts of the Marine Brown Alga Lobophora variegata Inhibit HIV-1 Infection at the Level of Virus Entry into Cells
Source: PLoS One. 2014 Aug 21;9(8):e103895. doi: 10.1371/journal.pone.0103895 (PMC4140698; doi:10.1371/journal.pone.0103895)
Supplement: File S1 — Figure S1 and supplemental experimental procedures. Figure S1: Time-dependent effect of the L. variegata aqueous extract on the viability of Hela cells. A time series of the effect of the L. variegata aqueous extract on viability of Hela cells was determined by a colorimetric XTT assay. Hela cells were treated with a fixed concentration of 400 µg/ml of the extract for time periods of one day up to 7 days. Data are given relative to untreated cells measured at the same time point (100%). (DOCX) [file pone.0103895.s001.docx]

**Supporting information**

Figure S1: Time-dependent effect of the *L. variegata* aqueous extract on the viability of Hela cells. A time series of the effect of the *L. variegata* aqueous extract on viability of Hela cells was determined by a colorimetric XTT assay. Hela cells were treated with a fixed concentration of 400 µg/ml of the extract for time periods of one day up to 7 days. Data are given relative to untreated cells measured at the same time point (100%).


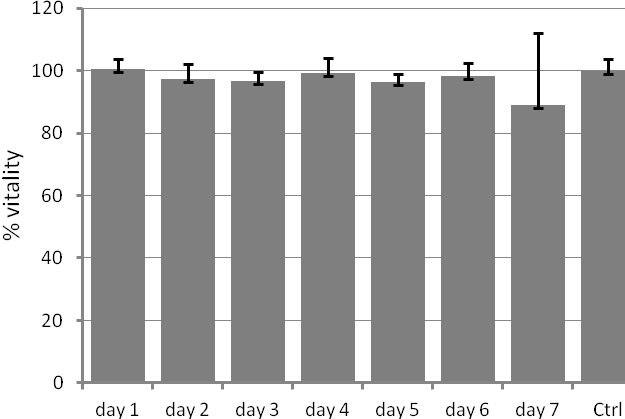


**Supplemental experimental procedures**

Hela cells were seeded into a 96-well plate (µCLEAR-Plate Black; Greiner Bio-One, Kremsmuenster, Germany) at a density of 2000 cells per well at 24 h prior to treatment. *L. variegata* aqueous extract was tested at a fixed concentration of 400 µg/ml in triplicates. Cells were incubated for up to 7 days after extract addition under standard conditions at 37 °C in 5% CO_2_. Cell culture medium was carefully replaced every second day with pre-warmed medium including fresh *L. variegata* aqueous extract. Viability of Hela cells was determined by a standard XTT test (TACS^®^ XTT Cell Proliferation Assay, Trevigen, USA) at various time points after extract addition (from one day up to 7 days). Cells were incubated with 0.3 mg/ml XTT working solution in 100 µl of culture medium for 2 h under standard culture conditions. XTT formazan concentrations was determined by an ELISA plate reader (SpectraMax M2 Multi-Mode Microplate Reader, Molecular Devices, USA) at a test wavelength of 490 nm and a reference wavelength of 630 nm. Values for treated cultures were related to those of untreated cultures in the same plate and the same length of incubation.
